# Proteomic analysis of the second-generation merozoites of *Eimeria tenella* under nitromezuril and ethanamizuril stress

**DOI:** 10.1186/s13071-019-3841-9

**Published:** 2019-12-18

**Authors:** Xue-Yan Li, Li-Li Liu, Min Zhang, Li-Fang Zhang, Xiao-Yang Wang, Mi Wang, Ke-Yu Zhang, Ying-Chun Liu, Chun-Mei Wang, Fei-Qun Xue, Chen-Zhong Fei

**Affiliations:** Key Laboratory of Veterinary Chemical Drugs and Pharmaceutics, Ministry of Agriculture and Rural Affairs, Shanghai Veterinary Research Institute, Chinese Academy of Agricultural Sciences, Shanghai, 200241 People’s Republic of China

**Keywords:** *Eimeria tenella*, Second-generation merozoites, Nitromezuril, Ethanamizuril, Label-free quantification proteomics

## Abstract

**Background:**

*Eimeria tenella* is a highly pathogenic coccidian that causes avian coccidiosis. Both nitromezuril (NZL) and ethanamizuril (EZL) are novel triazine compounds with high anticoccidial activity, but the mechanisms of their action are still unclear. This study explored the response of *E. tenella* to NZL and EZL by the study of changes in protein composition of the second-generation merozoites.

**Methods:**

Label-free quantification (LFQ) proteomics of the second-generation merozoites of *E. tenella* following NZL and EZL treatment were studied by LC-MS/MS to explore the mechanisms of action. The identified proteins were annotated and analyzed by Gene Ontology (GO), Kyoto Encyclopedia of Genes and Genomes (KEGG) pathway analysis and protein-protein interaction (PPI) networks analysis.

**Results:**

A total of 1430 proteins were identified by LC-MS/MS, of which 375 were considered as differential proteins in response to drug treatment (DPs). There were 26 only found in the NZL treatment group (N-group), 63 exclusive to the EZL treatment group (E-group), and 80 proteins were present in both drug groups. In addition, among the DPs, the abundant proteins with significantly altered expression in response to drug treatment (SDPs) were found compared with the C-group, of which 49 were upregulated and 51 were downregulated in the N-group, and 66 upregulated and 79 downregulated in the E-group. Many upregulated proteins after drug treatment were involved in transcription and protein metabolism, and surface antigen proteins (SAGs) were among the largest proportion of the downregulated SDPs. Results showed the top two enriched GO terms and the top one enriched pathway treated with EZL and NZL were related, which indicated that these two compounds had similar modes of action.

**Conclusions:**

LFQ proteomic analysis is a feasible method for screening drug-related proteins. Drug treatment affected transcription and protein metabolism, and SAGs were also affected significantly. This study provided new insights into the effects of triazine anticoccidials against *E. tenella*.
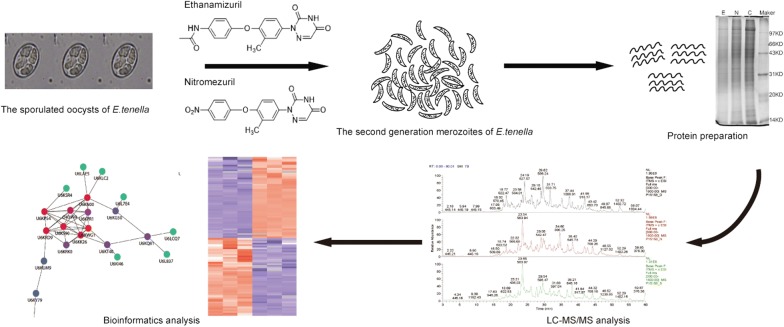

## Background

Coccidiosis is a serious intestinal disease caused by *Eimeria* (Apicomplexa: Eimeriidae) with strict host specificity and results in huge economic losses in the global poultry industry every year [1, 2]. *Eimeria tenella* is considered to be a highly pathogenic and prevalent, and was selected as the candidate for study. The development of parasites in host cells includes asexual and sexual reproduction, and the main part of the endogenous phase is merogony. During asexual reproduction, trophozoites undergo multiple divisions to form schizonts, which further generate numerous merozoites. A large increase in the second-generation merozoites of coccidia causes severe damage to the intestinal mucosa, leading to fatal hematogenous dysentery. Therefore, it is probably a wise choice to study the changes of the second-generation merozoites after drug treatment.

The control of coccidiosis principally depends on prophylactic chemotherapy by the inclusion of anticoccidials in feed. However, extensive drug use has led to the emergence of drug-resistant strains of coccidia. Hence, there is an urgent need to find new drugs or control strategies to deal with the development of drug resistance [3–5]. Over the years, triazine anticoccidial drugs have been used in the veterinary community worldwide to combat protozoan parasites [6]. Toltrazuril and diclazuril, the representatives of triazines, are effective across the entire endogenous phase of *Eimeria* [7]. Nitromezuril (NZL) and ethanamizuril (EZL) are relatively new triazine anticoccidial compounds. Previous studies found that NZL had high effectiveness against coccidiosis in broiler chickens at a dosage of 3 mg/kg in feed and little cross-resistance with diclazuril. EZL also exhibited similar high anticoccidial activity at a dosage of 10 mg/kg in feed [8]. As a result of EZL treatment, the differentiation of the second-generation schizonts and microgamonts, the shape of merozoites, the formation of oocyst wall and zygotes were affected in varying degrees, and mRNA expression and translation of enolase were downregulated [9, 10]. However, the molecular mechanisms of action of NZL and EZL are not clear yet.

Proteomics analysis has provided an in-depth understanding of cellular processes of specific organisms and served as a basis for screening specific molecular markers of drug action [11]. The proteomes of four life stages of *E. tenella* (unsporulated oocysts, sporulated oocysts, sporozoites and second-generation merozoites) were extensively studied using a MudPIT shotgun approach and two-dimensional electrophoresis, which found that a greater abundance of proteins in merozoites than sporozoites were linked to transcription, protein synthesis and cell cycle [12, 13]. The rhoptry proteome of sporozoites of *E. tenella* was investigated and different classes of rod-like proteins were identified, most of which had different degrees of homology with that of *Toxoplasma gondii* and *Neospora caninum* proteins and almost no homology with other known coccidial proteins [14]. The proteins of the second-generation merozoites of *E. tenella* expressed in response to drug treatment by diclazuril were analyzed and identified, 13 of which were involved in invasion, metabolism and surface antigens [15]. The effect of diclazuril on Hsp90 in the second-generation merozoites provided an insight into the molecular mechanism of diclazuril [16].

In the present study, LFQ proteomics methods were used to investigate the effect on the proteome of the second-generation merozoites of *E. tenella* treated with NZL and EZL. The possible targets and mechanisms of action are discussed according to the effects of NZL and EZL on the *E. tenella* proteome, which provides a foundation for further studying the molecular mechanisms of action of anticoccidial drugs.

## Methods

### Drugs and parasites

NZL and EZL (purity > 98%) were provided by the Shanghai Veterinary Research Institute, Chinese Academy of Agriculture Sciences. The strain used was a sensitive strain of *E. tenella* (CAAS2111606), which was isolated from a chicken farm (Shanghai, China), and has been retained in the Key Laboratory of Animals Parasitology of the Ministry of Agriculture and Rural Affairs since 1993. *Eimeria tenella* was propagated in coccidia-free chickens, and sporulated oocysts were stored in 2.5% potassium dichromate at 4 °C to maintain their viability and rejuvenated before infection.

### Chickens

180 one-day-old healthy Pudong yellow broiler chickens were purchased from Minyou Poultry Breeding Cooperative (Shanghai, China), and reared in a coccidia-free environment and provided with feed and water *ad libitum*. At two weeks of age, chickens were randomly divided into three groups of 60 chickens each, and each group was subdivided into three subgroups of 20 chickens each as biological replicates. Three groups were the NZL treatment group (N-group), the EZL treatment group (E-group) and the non-medicated control group (C-group), respectively. All chickens were challenged by oral gavage with a single dose of 8 × 10^4^ *E. tenella* sporulated oocysts and provided with standard diet without drug supplements. The chickens in the E-group and N-group were respectively given 15 mg/kg EZL and 5 mg/kg NZL at 96 h post-infection, while the chickens in the C-group did not receive any treatments.

### Extraction and purification of the second-generation merozoites

At 120 h after infection, all birds were sacrificed under carbon dioxide anesthesia and the ceca were harvested. The second-generation merozoites of *E. tenella* were extracted and purified according to the previous procedure [17]. Briefly, the ceca were dissected longitudinally with scissors and the inner wall of the cecum were with rinsed with ice-cold PBS (pH 7.4) to remove intestinal contents. Then the ceca were chopped up with scissors, and digested with trypsin (0.5 µg/ml) for 30–45 minutes at 37 °C. Finally, merozoites were purified using a 300-mesh sieve for filtration, centrifugation, erythrocytes disruption and percoll density gradient centrifugation. The purified merozoites precipitation was aliquoted and stored at – 80 °C until use.

### Protein samples preparation and trypsin digestion

Protein extraction and digestion were performed according to a slightly modified filter-aided sample preparation (FASP) method. Each sample of the second-generation merozoites was added to 400 μl lysis buffer (20 mM HEPES, 9 M Urea, pH 8.0), ground with a grinding rod until homogeneous. The samples were further sonicated on ice at 80 W, ultrasound time 10 s, rest time 15 s, and the sonication process repeated 10 times. Cell debris was then removed by centrifugation at 14,000×*g* for 30 min, and the supernatants were transferred into a new tube. The protein concentration was determined using the Bradford method. After quantification, protein samples were adjusted to the same concentration (20 µg/tube) for SDS-PAGE analysis.

Approximately 200 µg protein sample from each subgroup was reduced with dithiothreitol (DTT, final concentration 10 mM) for 1 h at 37 °C followed by alkylation with indoleacetic acid (IAA, final concentration 20 mM) for 30 min at room temperature in the dark. Sample digestion was performed in 120 µl ammonium bicarbonate (50 mM) with trypsin (enzyme to protein ratio of 1:25 w/w; Promega, Madison, USA), rocked at 600× *rpm* for 1 min and incubated at 37 °C for 16–18 h. After digestion, 0.25% trifluoroacetic acid in water was added into the mixture to hydrolyze the trypsin. The peptide mixture was then desalted using a C_18_ Cartridge (Sigma-Aldrich, Darmstadt, Germany).

### LC-MS/MS analysis

The peptides were analyzed by nanoflow liquid chromatography tandem mass spectrometry (LC-MS/MS) using a Q Exactive plus mass spectrometer (Thermo Fisher Scientific, Waltham, USA). The LC system was equipped with analytical EASY column SC200 (150 μm × 100 mm, RP-C_18_; Thermo Fisher Scientific) and a trap EASY column SC001 (150 µm × 20 mm, RP-C_18_; Thermo Fisher Scientific). Five μg of sample was loaded through an automatic sampler. For peptide separation, a gradient protocol of liquid chromatographic conditions was implemented. Solvent A was 2% acetonitrile in water containing 0.1% formic acid. Solvent B was 84% acetonitrile in water containing 0.1% formic acid. The gradient was used in a single run: first the column was balanced with 100% solvent A, followed by a linear gradient of 0–45% solvent B (100 min), and then 45–100% solvent B (8 min), finally 100% solvent B (12 min) at constant flow rate of 0.3 ml/min. The entire instrument was operated in positive mode for 120 min with the scan range of 300–1800 m/z and nominal mass resolving power of 70,000. Rapid, automated data-dependent capabilities enabled real-time acquisition of high-mass accuracy MS/MS spectra per 40 ms.

### Protein identification and quantification

The raw MS data processing, protein identification and relative quantitative analysis of samples were all performed using MaxQuant software (version 1.3.0.5) [18]. Proteins were distinguished by searching protein sequences in the Uniprot *Eimeria tenella* database. MaxQuant search parameters were as follows: MS/MS tolerance, 0.1 Da; peptide mass tolerance, 20 ppm; enzyme, trypsin; the number of missed cleavages, 2; fixed modification, carbamidomethyl (C); variable modification, oxidation (M) and Acetyl (Protein N-term). Two peptides (at least one unique and unmodified) matching the same proteins were required for protein identification at a maximum false discovery rate (FDR) of ≤ 0.01. Unique and razor peptides were used for peptide assignments and protein quantification (Additional file 1: Table S1). Peptides were matched across different MS/MS runs based on mass and retention time (match between runs of 2 min) (Additional file 2: Table S2). To minimize technical variations and compare the abundances of the same proteins (treated and non-treated with each drug), the average LFQ of three biological replicates were used. To determine up and downregulated proteins (treated *vs* non-treated with each drug), a Studentʼs t-test was performed on LFQ intensity using MaxQuant’s Perseus software (Additional file 3: Tables S3, S4, S5, S6). All abundance ratios gained from proteomic experiments were log_2_-transformed and the median normalized to zero.

### Bioinformatics analysis

Blast2GO was used to classify these identified proteins by Gene Ontology (GO) (http://www.geneontology.org) including three biological aspects, biological process, molecular function and cellular component. GO terms indicated that proteins were divided into different functional classes. To correctly place DPs within signaling pathways, Kyoto Encyclopedia of Genes and Genomes (KEGG) (http://www.kegg.jp/kegg/) was used to predict molecular function, biological processes and pathways. Protein-protein interaction (PPI) networks were constructed using the Search Tool of the Retrieval of Interaction Genes/Proteins (STRING) database, in which PPI of DPs (combined score > 0.9) was illustrated by Cytoscape software to further analyze the interaction networks.

### Statistical analysis

Statistical differences between samples were assessed using Studentʼs t-test, and considered to be statistically significant at *P* *<* 0.05.

## Results

### Label-free quantification proteomic analysis

**Fig. 1:**
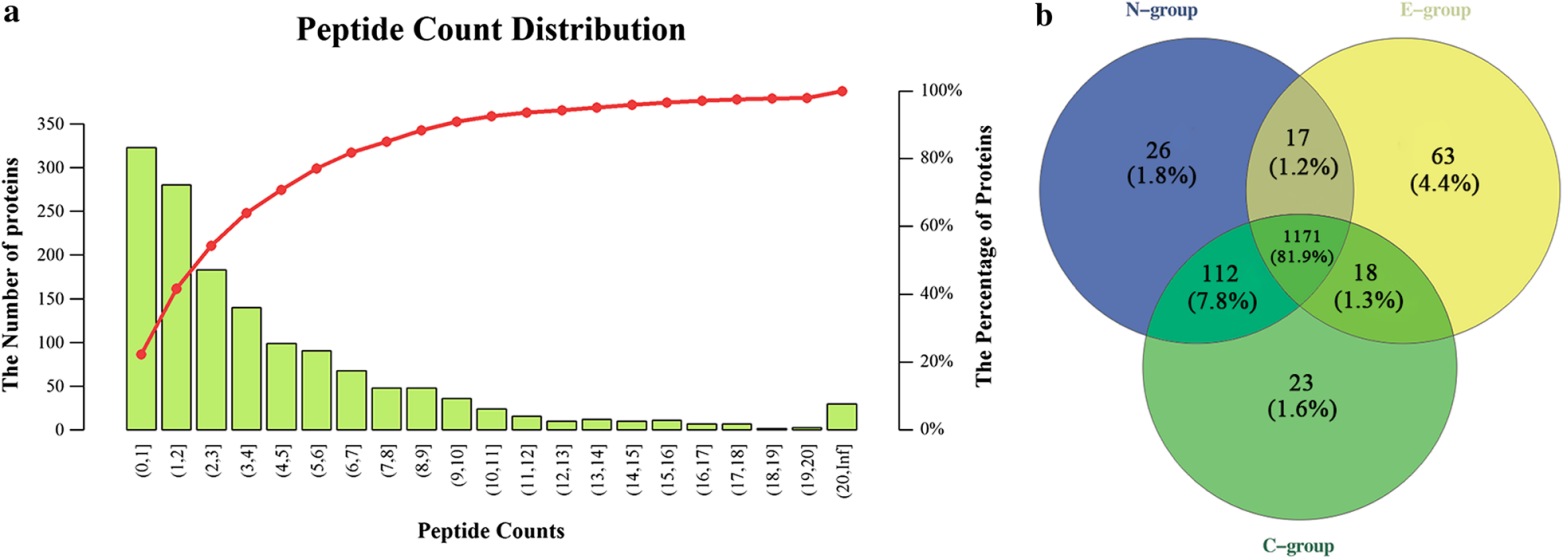
LC-MS/MS analysis of groups. **a** Peptide count distribution of identified proteins. **b** Area-proportional Venn diagram of the identified proteins among N-, E- and C-group

**Fig. 2:**
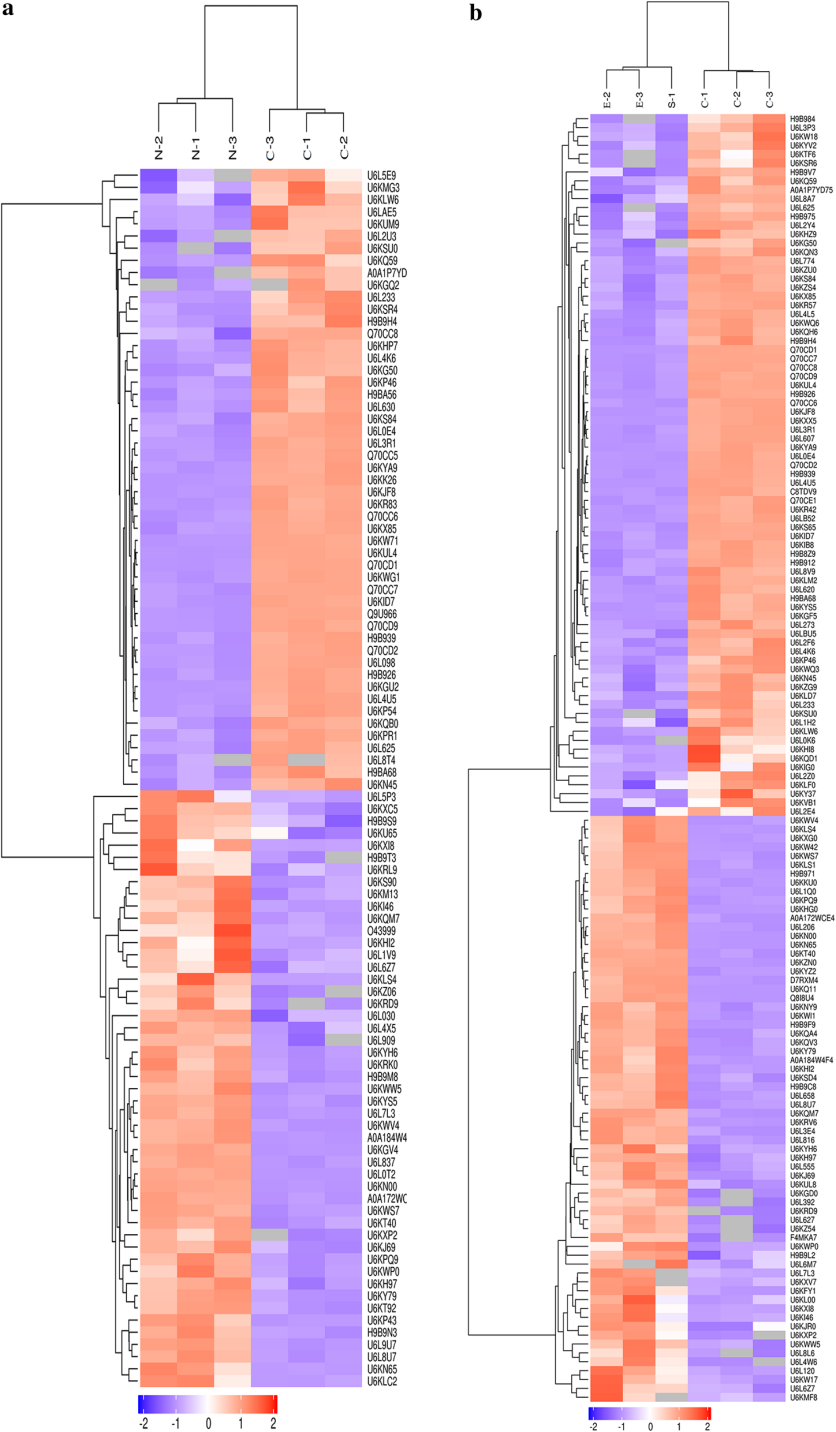
Hierarchical clustering analysis of SDPs. **a** Hierarchical clustering analysis of SDPs between the N-group (N) and C-group (C). **b** Hierarchical clustering analysis of SDPs between the E-group (E) and C-group (C). Every trial was performed in triplicate and the experiment was carried out twice

The counting distribution of distinct peptide sequences in mass spectrometry analysis is shown in Fig. 1a. A total of 1430 proteins of the second-generation merozoites of *E. tenella* were identified in three groups. A total of 375 DPs were identified, 26 found only in the N-group, 63 exclusive to the E-group, 23 unique in the C-group, and there were 80 DPs found in both the N-group and E-group (Fig. 1b). Compared with proteins in the C-group, 100 SDPs were found in the N-group, of which 49 were upregulated and 51 were downregulated (Fig. 2a, Additional file 4: Table S7). In the E-group, 66 upregulated and 79 downregulated SDPs were found (Fig. 2b, Additional file 4: Table S8). Many upregulated proteins resulting from drug treatment were affected in transcription and protein metabolism, such as helicase (UniProt: U6KXI8), phosphotransferase (UniProt: A0A172WCE4), glycyl-tRNA synthetase (UniProt: U6L8U7), isoleucine-tRNA synthetase (UniProt: U6KPQ9), and elongation factor 2 (UniProt: A0A184W4F4). Several SAG family members, such as SAG13 (UniProt: Q70CD1), SAG family number (UniProt: U6L4U5), SAG4 (UniProt: Q70CD9), SAG18 (UniProt: Q70CC6) were apparently downregulated with drug treatment. Almost half of DPs were described as unannotated proteins, which are worthy of further study.

### GO annotations and KEGG pathway analysis

**Fig. 3:**
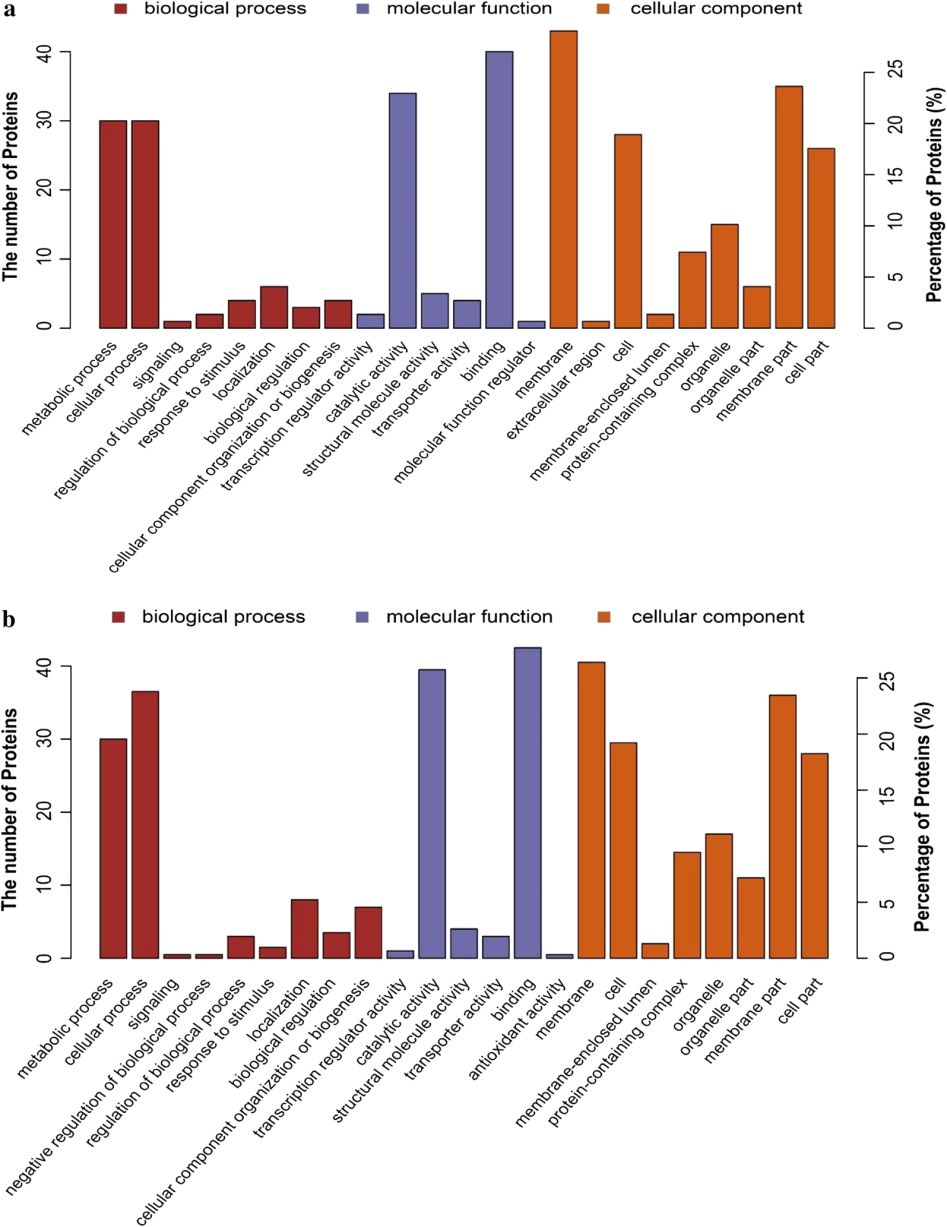
GO enrichment analysis of DPs. **a** GO enrichment analysis of DPs in the N-group. **b** GO enrichment analysis of DPs in the E-group. Biological process: the top nine enriched GO terms; Molecular function: the top six enriched GO terms; Cellular components: the top nine enriched GO terms in the N-group/the top eight enriched GO terms in the E-group

DPs were divided into three classes including biological process, molecular function and cellular component based on the GO classification system and UniProt database. The number and percentage of DPs in the main subcategories of GO terms are shown in Fig. 3. After drug action, most of the DPs were mainly associated with metabolic and cellular processes. The proteins of molecular function class were mainly involved in binding, catalytic activity and outer membrane. For the cellular component, most proteins were distributed in the membrane part and the cellular part. The most enriched GO terms of the E-group were similar with those of the N-group except for the extracellular region.

**Fig. 4:**
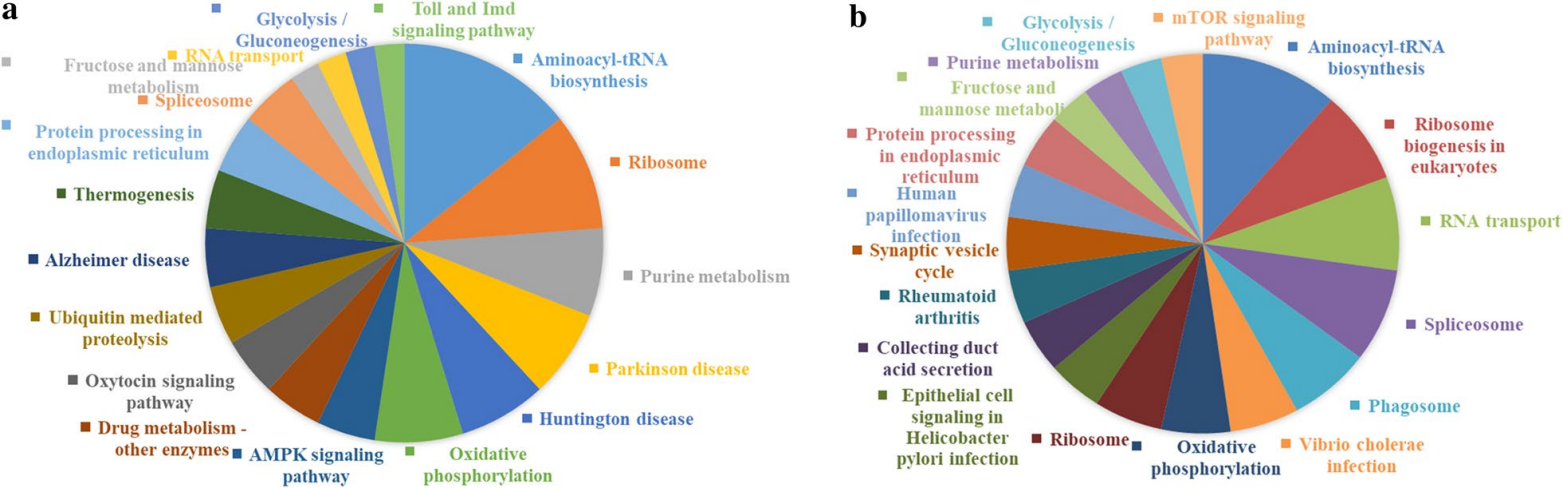
KEGG pathways analysis of DPs. **a** Top 18 enriched KEGG pathway analysis result of the N-group. DPs in the N-group involved in 72 pathways and almost closely related to aminoacyl-tRNA biosynthesis, ribosome and purine metabolism. **b** Top 18 enriched KEGG pathway analysis result of the E-group. DPs in the E-group covered 94 pathways and mainly referred to aminoacyl-tRNA biosynthesis, ribosome biogenesis in eukaryotes and RNA transport

DPs were mapped to the reference pathway in the KEGG database to identify the parasite biological pathway under the action of NZL and EZL. The top eighteen enriched KEGG pathways of DPs were shown in Fig. 4. Among DPs in the N-group, only 45 DPs (14 downregulated and 31 upregulated) were involved in 72 pathways in KEGG pathway analysis. These top eighteen enriched pathways were almost closely related to aminoacyl-tRNA biosynthesis, ribosome and purine metabolism (Fig. 4a). KEGG pathway analysis that revealed DPs in the E-group covered 94 pathways, and the top eighteen enriched pathways included aminoacyl-tRNA biosynthesis, ribosome biogenesis in eukaryotes and RNA transport (Fig. 4b).

### PPI network analyses of DPs

**Fig. 5:**
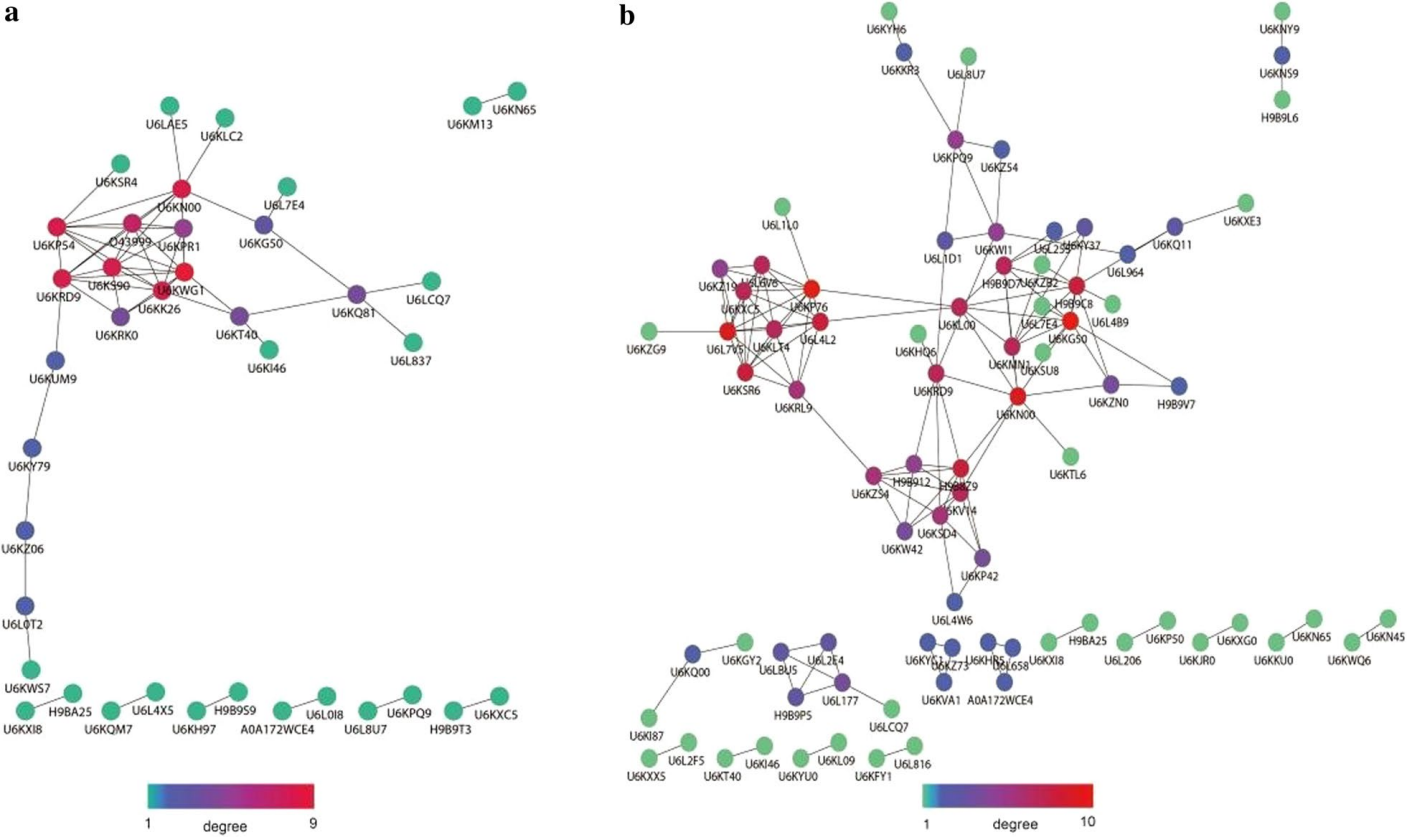
Protein-protein interaction (PPI) network analyses. **a** PPI network analysis result for the N-group. **b** PPI network analysis result for the E-group. Colored nodes indicate the various degrees of proteins interaction evidence. Average node degree: 2.8 in the N-group; and 3.2 in the E-group

PPI network analyses of DPs were constructed using STRING 10 and visualized with Cytoscape. Thirty-eight DPs in the N-group and 81 DPs in the E-group matched PPI networks, respectively. Every protein interacted with at least one other partner. Proteins located in the connected nodes with higher connectivity than others were referred to as “hubs”, which played a crucial role in PPI network analyses. Colored nodes indicated the various degrees of protein interaction evidence (Average node degree: 2.8 in the N-group and 3.2 in the E-group) (Fig. 5). In the N-group, six proteins (UniProt: U6KWG1, U6KP54, U6KRD9, U6KK26, U6KS90 and U6KN00) presented the highest degree of connectivity (≥ 8 edges) (Fig. 5a). In the E-group, there were eight proteins which were similar to RNA binding motif-containing protein (UniProt: U6KG50), WD-40 repeat protein (UniProt: U6KP76), elongation factor 2 (UniProt: U6KN00), uncharacterized protein (UniProt: U6L7V5), glutamyl-tRNA synthetase (UniProt: U6L4L2), 40S ribosomal protein SA (UniProt: H9B8Z9), ribosome biogenesis regulatory protein (UniProt: U6KSR6), peptidylprolyl isomerase (UniProt: H9B9C8) (≥ eight edges) were found to be hubs in functional interaction network (Fig. 5b).

## Discussion

Anticoccidial drugs may achieve anticoccidial effect by regulating the abundance of functional proteins of coccidia. NZL and EZL are two new triazine anticoccidial compounds developed in recent years. Proteomic studies can provide important information about the effects of drugs on organisms.

LFQ proteomics of the second-generation merozoites of *E. tenella* treated with NZL and EZL was conducted and 375 DPs were identified (148 in the N-group and 307 in the E-group), many of which were unannotated proteins (55 in the N-group and 144 in the E-group). These DPs were mostly related to translation and membrane proteins. The top two downregulated proteins were SAG family members, and an uncharacterized protein. There were 21 and 20 downregulated DPs belonging to SAG family members in the N-group and E-group, respectively, including SAG13 (UniProt: Q70CD1), SAG21 (UniProt: Q70CC8), SAG4 (UniProt: Q70CD9), SAG18 (UniProt: Q70CC6), SAG family member (UniProt: U6L233), which were shared by two groups. It was reported that 89 SAGs are located on the membrane surface of *E. tenella* [19]. SAGs are the principal surface antigens of *E. tenella*, and encoding a single domain of membrane binding proteins tethered by glycosylphosphatidylinositol (GPI)-anchors to the surface of invasive sporozoites and merozoites [20]. Most SAGs were expressed in the second-generation merozoites in a time dependent fashion, which might be related to host cell adhesion, but their specific biological functions are still unclear [12, 21, 22]. The invasion of sporozoites of *E. tenella* could also be inhibited significantly by interaction with *Et*SAG1 monoclonal antibody 2H10E3 [23]. Interestingly, the damage to the membrane structure of *E. tenella* could be clearly observed under the action of EZL [9]. After NZL and EZL treatment, SAGs were significantly downregulated. It was presumed that drug action decreased the abundance of SAGs, further damaging the integrity of the membrane protein structure. The mechanism of drug action on SAGs deserves further study.

GO enrichment and KEGG pathway analysis showed that the majority of DPs were related to transcription and protein synthesis. The top two enriched GO terms of three classes and the top one enriched pathway between the E-group and N-group were similar, indicating the similarity of the modes of action of these two compounds (Figs. 3, 4). These important proteins including helicase (UniProt: U6KXI8), RNA binding protein (UniProt: U6KH97), aminoacyl-tRNA synthetases (UniProt: U6KPQ9, U6L8U7, U6L6Z7, U6KNU0 and U6KNU0), which were upregulated and play important roles in transcription and protein synthesis in both drug groups. Helicase is an essential protein for effective and accurate replication, repair, and recombination of genomes, which facilitated RNA metabolic processes such as transcription, ribosome biogenesis, translation and RNA splicing [24]. RNA binding proteins act on post-transcriptional regulation of RNA. Aminoacyl-tRNA synthetase binds to specific tRNA of amino acids required for ribosomal protein synthesis and is considered to be an important housekeeping enzyme in protein homeostasis [25]. Studies have shown that aminoacyl-tRNA synthetase as drug target could effectively inhibit the growth of *T. gondii*, *Cryptosporidium* and *Plasmodium* [25, 26].

Thirty-eight DPs in the N-group and 81 DPs in the E-group were matched by PPI functional networks, respectively (Fig 5). There were some proteins (UniProt: U6KWG1, U6KP54, U6KRD9, U6KK26, U6KS90 and U6KN00) in the N-group referred as the top six hub proteins, which are important proteins that are seemingly networked more than others. These hub proteins may therefore represent important proteins that affect various pathways in *E. tenella* [27–29]. Eight proteins (UniProt: U6KG50, U6KP76, U6KN00, U6L7V5, U6L4L2, H9B8Z9, U6KSR6 and H9B9C8) in the E-group were found to be hubs in the connected nodes of PPI, which mainly exhibited important roles in transcription [30–32]. The present study provided new insights into the mechanisms of action of NZL and EZL against *E. tenella*. The upregulated DPs mainly related to protein synthesis and the downregulated SAGs were important components of the membrane structure of *E. tenella*, which might be helpful to better understand the action mechanism and resistance mechanism of triazine anticoccidials.

## Conclusions

The LFQ proteomics method was used to analyze the second-generation merozoites of *E. tenella* treated and non-treated with NZL and EZL in order to explore the response of *E. tenella* to these drugs. Many identified DPs were involved in pathways related to transcription and protein synthesis, most of which were upregulated under NZL and EZL treatment. Interestingly, SAGs were among the largest proportion of downregulated DPs. This study implied that these two drugs probably have a similar mechanism of action. Many proteins identified in this study provide a new insight into the targets of triazine anticoccidial drugs against *E. tenella*.

## Supplementary information

**Additional file 1: Table S1.** Protein identification of the second-generation merozoites of *E. tenella* (non-treated and treated with NZL/EZL). *Abbreviations*: C, C-group; N, N-group; E, E-group.

**Additional file 2: Table S2.** Peptide identification by LC-MS/MS analysis of the second-generation merozoites of *E. tenella*. *Abbreviations*: C, C-group; N, N-group; E, E-group.

**Additional file 3: Table S3.** Protein quantification and difference analysis of the second-generation merozoites of *E. tenella*. **Table S4.** The significant differences in analysis of proteins of the second-generation merozoites of *E. tenella* treated with NZL. **Table S5.** Protein quantification and differences in analysis of the second-generation merozoites of *E. tenella*. **Table S6.** The significant differences in analysis of proteins of the second-generation merozoites of *E. tenella* treated with EZL. *Abbreviations:* C, C-group; N, N-group; E, E-group.

**Additional file 4: Table S7.** SDPs of the second-generation merozoites of *E. tenella* treated with NZL. **Table S8.** SDPs of the second-generation merozoites of *E. tenella* treated with EZL.

## Data Availability

The mass spectrometry proteomics data (Project Name: Proteomic analysis of the second-generation merozoites of *Eimeria tenella* under nitromezuril and ethanamizuril stress; project accession: PXD016177) have been deposited to the ProteomeXchange Consortium *via* the PRIDE partner repository [33].

## Abbreviations

NZL: nitromezuril
EZL: ethanamizuril
N-group: NZL treatment group
E-group: EZL treatment group
C-group: non-medicated control group
LFQ: label-free quantification
DPs: differential proteins in response to drug treatment
SDPs: abundant proteins with significantly altered expression in response to drug treatment
GO: Gene Ontology
KEGG: Kyoto Encyclopedia of Gene and Genomes
PPI: protein–protein interaction
PBS: phosphate buffered saline
SDS-PAGE: sodium dodecyl sulfate polyacrylamide gel electrophoresis
DTT: dithiothreitol
IAA: indoleacetic acid
FASP: filter-aided sample preparation
LC-MS/MS: liquid chromatography tandem mass spectrometry
FDR: false discovery rate

## References

[1] Williams RB (1999). A compartmentalised model for the estimation of the cost of coccidiosis to the world’s chicken production industry. Int J Parasitol..

[2] Chapman HD, Barta JR, Blake D, Gruber A, Jenkins M, Smith NC (2013). A selective review of advances in coccidiosis research. Adv Parasitol..

[3] Witcombe DM, Smith NC (2014). Strategies for anti-coccidial prophylaxis. Parasitology..

[4] Blake DP, Tomley FM (2014). Securing poultry production from the ever-present *Eimeria* challenge. Trends Parasitol..

[5] Matsubayashi M, Hatta T, Miyoshi T, Anisuzzaman, Alim MA, Yamaji K (2012). Synchronous development of *Eimeria tenella* in chicken caeca and utility of laser microdissection for purification of single stage schizont RNA. Parasitology..

[6] Stock ML, Elazab ST, Hsu WH (2018). Review of triazine antiprotozoal drugs used in veterinary medicine. J Vet Pharmacol Ther..

[7] Laczay P, Voros G, Semjen G (1995). Comparative studies on the efficacy of sulphachlorpyrazine and toltrazuril for the treatment of caecal coccidiosis in chickens. Int J Parasitol..

[8] Zhang M, Li X, Zhao Q, She R, Xia S, Zhang K (2019). Anticoccidial activity of novel triazine compounds in broiler chickens. Vet Parasitol..

[9] Liu L, Chen H, Fei C, Wang X, Zheng W, Wang M (2016). Ultrastructural effects of acetamizuril on endogenous phases of *Eimeria tenella*. Parasitol Res..

[10] Liu LL, Chen ZG, Mi RS, Zhang KY, Liu YC, Jiang W (2016). Effect of acetamizuril on enolase in second-generation merozoites of *Eimeria tenella*. Vet Parasitol..

[11] Gozal YM, Duong DM, Gearing M, Cheng D, Hanfelt JJ, Funderburk C (2009). Proteomics analysis reveals novel components in the detergent-insoluble subproteome in Alzheimer’s disease. J Proteome Res.

[12] Lal K, Bromley E, Oakes R, Prieto JH, Sanderson SJ, Kurian D (2009). Proteomic comparison of four *Eimeria tenella* life-cycle stages: unsporulated oocyst, sporulated oocyst, sporozoite and second-generation merozoite. Proteomics..

[13] Liu L, Liu YS, Liu GH, Cheng TY (2018). Proteomics analysis of faecal proteins in the tick *Haemaphysalis flava*. Parasites Vectors..

[14] Oakes RD, Kurian D, Bromley E, Ward C, Lal K, Blake DP (2013). The rhoptry proteome of *Eimeria tenella* sporozoites. Int J Parasitol..

[15] Shen XJ, Li T, Fu JJ, Zhang KY, Wang XY, Liu YC (2014). Proteomic analysis of the effect of diclazuril on second-generation merozoites of *Eimeria tenella*. Parasitol Res..

[16] Shen X, Wang C, Zhu Q, Li T, Yu L, Zheng W (2012). Effect of the diclazuril on Hsp90 in the second-generation merozoites of *Eimeria tenella*. Vet Parasitol..

[17] Zhou B, Wang H, Xue F, Wang X, Fei C, Wang M (2010). Effects of diclazuril on apoptosis and mitochondrial transmembrane potential in second-generation merozoites of *Eimeria tenella*. Vet Parasitol..

[18] Bielow C, Mastrobuoni G, Kempa S (2016). Proteomics quality control: quality control software for MaxQuant results. J Proteome Res..

[19] Reid AJ, Blake DP, Ansari HR, Billington K, Browne HP, Bryant J (2014). Genomic analysis of the causative agents of coccidiosis in domestic chickens. Genome Res..

[20] Tabarés E, Ferguson D, Clark J, Soon PE, Wan KL, Tomley F (2004). *Eimeria tenella* sporozoites and merozoites differentially express glycosylphosphatidylinositol-anchored variant surface proteins. Mol Biochem Parasitol.

[21] Belli SI, Walker RA, Flowers SA (2010). Global protein expression analysis in apicomplexan parasites: current status. Proteomics..

[22] Silmon de Monerri NC, Weiss LM (2015). Integration of RNA-seq and proteomics data with genomics for improved genome annotation in apicomplexan parasites. Proteomics..

[23] Jahn D, Matros A, Bakulina AY, Tiedemann J, Schubert U, Giersberg M (2009). Model structure of the immunodominant surface antigen of *Eimeria tenella* identified as a target for sporozoite-neutralizing monoclonal antibody. Parasitol Res..

[24] Patel SS, Donmez I (2006). Mechanisms of helicases. J Biol Chem..

[25] Jain V, Yogavel M, Kikuchi H, Oshima Y, Hariguchi N, Matsumoto M (2017). Targeting prolyl-tRNA synthetase to accelerate drug discovery against malaria, leishmaniasis, toxoplasmosis, cryptosporidiosis, and coccidiosis. Structure..

[26] Palencia A, Liu RJ, Lukarska M, Gut J, Bougdour A, Touquet B (2016). *Cryptosporidium* and *Toxoplasma* parasites are inhibited by a benzoxaborole targeting leucyl-tRNA synthetase. Antimicrob Agents Chemother..

[27] Kaul G, Pattan G, Rafeequi T (2011). Eukaryotic elongation factor-2 (eEF2): its regulation and peptide chain elongation. Cell Biochem Funct..

[28] Wang H, Zhao L, Li K, Ling R, Li X, Wang L (2006). Overexpression of ribosomal protein L15 is associated with cell proliferation in gastric cancer. BMC Cancer..

[29] Whelan NV, Halanych KM (2017). Who Let the CAT Out of the bag? Accurately dealing with substitutional heterogeneity in phylogenomic analyses. Syst Biol.

[30] Zhou X, Liao WJ, Liao JM, Liao P, Lu H (2015). Ribosomal proteins: functions beyond the ribosome. J Mol Cell Biol..

[31] Merritt EA, Arakaki TL, Gillespie JR, Larson ET, Kelley A, Mueller N (2010). Crystal structures of trypanosomal histidyl-tRNA synthetase illuminate differences between eukaryotic and prokaryotic homologs. J Mol Biol..

[32] Song DG, Kim YS, Jung BC, Rhee KJ, Pan CH (2013). Parkin induces upregulation of 40S ribosomal protein SA and posttranslational modification of cytokeratins 8 and 18 in human cervical cancer cells. Appl Biochem Biotechnol..

[33] Deutsch EW, Attila C, Zhi S, Andrew J, Yasset P-R, Tobias T, Campbell DS, Manuel B-L, Shujiro O, Shin K (2017). The proteomeXchange consortium in 2017: supporting the cultural change in proteomics public data deposition. Nucleic Acids Res..

