# Supplementary material for: Proteomic analysis of the second-generation merozoites of Eimeria tenella under nitromezuril and ethanamizuril stress
Source: Parasit Vectors. 2019 Dec 18;12:592. doi: 10.1186/s13071-019-3841-9 (PMC6921512; doi:10.1186/s13071-019-3841-9)
Supplement: Supplementary file 4 — Additional file 4: Table S7. SDPs of the second-generation merozoites of E. tenella treated with NZL. Table S8. SDPs of the second-generation merozoites of E. tenella treated with EZL. [file 13071_2019_3841_MOESM4_ESM.docx]

**Additional file 4: Table S7.** SDPs of the second-generation merozoites of *E. tenella* treated with NZL.

| Accession^a^ | Description | Fold change^b^ | *P* value^c^ |
| --- | --- | --- | --- |
| U6KGV4 | KH domain containing protein, putative | 12.55 | ＜0.0001 |
| U6KLS4 | Uncharacterized protein | 12.38 | 0.0107 |
| U6KXI8 | Helicase, putative | 9.66 | 0.0210 |
| U6L1V9 | Uncharacterized protein | 5.31 | 0.0241 |
| U6L7L3 | Uncharacterized protein | 5.27 | ＜0.0001 |
| O43999 | 40S ribosomal protein S3a | 5.20 | 0.0238 |
| U6KWW5 | Uncharacterized protein | 4.89 | 0.0004 |
| U6L909 | Exonuclease, putative | 4.73 | 0.0021 |
| U6KYH6 | Alanine--tRNA ligase | 4.21 | 0.0003 |
| U6KS90 | 60S acidic ribosomal protein P0 | 4.04 | 0.0020 |
| U6KI46 | Hypoxanthine phosphoribosyltransferase | 4.00 | 0.0047 |
| U6KU65 | Uncharacterized protein | 3.41 | 0.0364 |
| U6KRD9 | Carbonic anhydrase domain containing protein, putative | 3.10 | 0.0200 |
| H9B9T3 | Protein MAK16 homolog | 2.99 | 0.0464 |
| A0A172WCE4 | Phosphotransferase | 2.96 | ＜0.0001 |
| A0A184W4F4 | Elongation factor 2 | 2.93 | ＜0.0001 |
| U6KWP0 | YALI0F04037p, related | 2.87 | 0.0024 |
| U6KY79 | DnaJ domain-containing protein, putative | 2.82 | 0.0003 |
| U6KT40 | Inosine-5'-monophosphate dehydrogenase | 2.71 | 0.0001 |
| U6KHI2 | Uncharacterized protein | 2.69 | 0.0180 |
| U6KPQ9 | Isoleucine-tRNA synthetase, putative | 2.69 | 0.0005 |
| U6L8U7 | Glycyl-tRNA synthetase, putative | 2.69 | 0.0002 |
| U6L0T2 | Heat shock protein 90 | 2.68 | ＜0.0001 |
| H9B9N3 | Adenosine transporter, putative | 2.57 | 0.0007 |
| U6L4X5 | Uncharacterized protein | 2.53 | 0.0016 |
| U6KYS5 | Uncharacterized protein | 2.49 | ＜0.0001 |
| U6L837 | Acetyl-CoA acyltransferase B, putative | 2.45 | ＜0.0001 |
| U6KN00 | Elongation factor 2, putative | 2.45 | ＜0.0001 |
| U6L030 | Uncharacterized protein | 2.43 | 0.0028 |
| U6KLC2 | ATP-dependent RNA helicase, putative | 2.36 | 0.0075 |
| U6KWS7 | DnaJ domain-containing protein, putative | 2.33 | ＜0.0001 |
| U6KT92 | Uncharacterized protein | 2.27 | 0.0003 |
| U6L6Z7 | Histidyl-tRNA synthetase, putative | 2.25 | 0.0181 |
| U6KXP2 | Exportin, putative | 2.25 | 0.0048 |
| U6KJ69 | Uncharacterized protein | 2.25 | 0.0011 |
| U6KP43 | Hsc70/Hsp90-organizing protein, putative | 2.23 | 0.0008 |
| U6KWV4 | Opine dehydrogenase, putative | 2.23 | 0.0001 |
| U6KM13 | Inhibitor-1 of protein phosphatase type 2A, putative | 2.21 | 0.0043 |
| H9B9S9 | RNA binding motif-containing protein, putative | 2.18 | 0.0101 |
| U6L5P3 | Uncharacterized protein | 2.15 | 0.0343 |
| U6KZ06 | Uncharacterized protein | 2.13 | 0.0048 |
| U6KH97 | RNA binding protein, putative | 2.13 | 0.0016 |
| U6KQM7 | Ubiquitin-conjugating enzyme e2, putative | 2.09 | 0.0035 |
| U6KRK0 | Elongation factor 1, putative | 2.07 | 0.0010 |
| U6KXC5 | GTPase domain containing protein, putative | 2.06 | 0.0018 |
| U6KRL9 | Rna 3' terminal phosphate cyclase, putative | 2.05 | 0.0244 |
| H9B9M8 | Uncharacterized protein | 2.02 | 0.0002 |
| U6L9U7 | Threonyl-tRNA synthetase, putative | 2.02 | 0.0002 |
| U6KN65 | Adenosylhomocysteinase | 2.00 | 0.0034 |
| Q9U966 | Micronemal protein MIC4, related MIC4 | 0.50 | ＜0.0001 |
| U6KJF8 | Lysophospholipase, putative | 0.49 | ＜0.0001 |
| H9BA56 | Uncharacterized protein | 0.49 | 0.0002 |
| U6KX85 | Uncharacterized protein | 0.49 | ＜0.0001 |
| H9B9H4 | SAG family member | 0.49 | 0.0012 |
| U6KS84 | Uncharacterized protein | 0.48 | 0.0001 |
| U6KYA9 | Uncharacterized protein | 0.48 | ＜0.0001 |
| U6KUM9 | Heat shock protein, putative | 0.48 | 0.0030 |
| U6KQB0 | Uncharacterized protein | 0.48 | 0.0002 |
| U6KW71 | SAG family member | 0.48 | ＜0.0001 |
| U6L098 | Microneme protein 4 MIC | 0.48 | ＜0.0001 |
| H9B926 | SAG family member | 0.47 | ＜0.0001 |
| U6L630 | SAG family member | 0.47 | 0.0002 |
| U6KK26 | 60S ribosomal protein L13 | 0.47 | ＜0.0001 |
| U6KHP7 | Uncharacterized protein | 0.47 | 0.0001 |
| H9B939 | SAG family member (Sag22) | 0.46 | ＜0.0001 |
| Q70CC7 | SAG family member (Sag23) | 0.46 | ＜0.0001 |
| U6KSU0 | Uncharacterized protein | 0.44 | 0.0026 |
| U6KG50 | RNA binding motif-containing protein, putative | 0.44 | 0.0005 |
| U6KPR1 | Ribosomal protein L27, putative | 0.43 | 0.0001 |
| U6KID7 | Uncharacterized protein | 0.43 | ＜0.0001 |
| H9BA68 | SAG family member | 0.43 | 0.0005 |
| U6KMG3 | Zinc finger (CCCH type) protein, putative | 0.42 | 0.0248 |
| U6L5E9 | Peptidase M16 domain containing protein, putative | 0.41 | 0.0395 |
| U6KGQ2 | Uncharacterized protein | 0.41 | 0.0235 |
| U6KQ59 | Uncharacterized protein | 0.40 | 0.0019 |
| U6KR83 | Uncharacterized protein | 0.40 | ＜0.0001 |
| U6L3R1 | SAG family member | 0.40 | ＜0.0001 |
| U6L8T4 | Uncharacterized protein | 0.38 | 0.0157 |
| U6KP46 | Uncharacterized protein | 0.38 | 0.0007 |
| U6KSR4 | Nascent polypeptide-associated complex alpha chain | 0.38 | 0.0011 |
| Q70CC8 | SAG family member (Sag21) | 0.38 | 0.0016 |
| U6KUL4 | SAG family member | 0.37 | ＜0.0001 |
| Q70CD2 | SAG family member (Sag16) | 0.36 | ＜0.0001 |
| U6KP54 | 60S ribosomal protein L19, putative | 0.35 | ＜0.0001 |
| A0A1P7YD75 | Phosphodiesterase | 0.34 | 0.0007 |
| U6L233 | SAG family member | 0.33 | 0.0015 |
| U6L2U3 | Uncharacterized protein | 0.33 | 0.0034 |
| U6L625 | SAG family member | 0.33 | 0.0002 |
| U6KLW6 | Uncharacterized protein | 0.33 | 0.0086 |
| Q70CC6 | SAG family member (Sag18) | 0.32 | ＜0.0001 |
| U6L0E4 | Uncharacterized protein | 0.31 | ＜0.0001 |
| Q70CD9 | SAG family member (Sag4) | 0.29 | ＜0.0001 |
| U6L4K6 | Uncharacterized protein | 0.28 | 0.0001 |
| U6KN45 | Dynein intermediate chain, putative | 0.27 | 0.0004 |
| U6KWG1 | Ribosomal protein L15 | 0.26 | ＜0.0001 |
| U6L4U5 | SAG family member | 0.24 | ＜0.0001 |
| Q70CC5 | SAG family member (Sag20) | 0.22 | ＜0.0001 |
| U6LAE5 | Uncharacterized protein | 0.19 | 0.0018 |
| Q70CD1 | SAG family member (Sag13) | 0.07 | ＜0.0001 |
| U6KGU2 | SAG family member | 0.04 | ＜0.0001 |

^a^ Access was protein accession in the UniProt *Eimeria tenella* database;

^b^ Fold change was quantitative expression difference of proteins in response to NZL;

^c^ *P-*value <0.05 identified the significant expression difference of SDPs in the N-group compared with those in the C-group；*P-*value < 0.0001 indicated that the exact *P*-value was very small.

**Additional file 4: Table S8.**  SDPs of the second-generation merozoites of *E. tenella* treated with EZL.

| Accession^a^ | Description | Fold change^b^ | *P-*value^c^ |
| --- | --- | --- | --- |
| U6KLS4 | Uncharacterized protein | 7.86 | 0.0005 |
| U6KXI8 | Helicase, putative | 5.56 | 0.0163 |
| U6KXV7 | 3-demethylubiquinone-9 3-methyltransferase, putative | 5.53 | 0.0005 |
| U6KRV6 | Uncharacterized protein | 5.24 | 0.0001 |
| U6KNY9 | Chromosome condensation protein, putative | 4.66 | 0.0004 |
| U6KT40 | Inosine-5'-monophosphate dehydrogenase | 4.27 | ＜0.0001 |
| U6KW17 | Uncharacterized protein | 3.88 | 0.0137 |
| Q8I8U4 | Lactate dehydrogenase | 3.82 | ＜0.0001 |
| U6KPQ9 | Isoleucine-tRNA synthetase, putative | 3.79 | 0.0003 |
| U6L206 | Peroxidoxin 2, putative | 3.56 | ＜0.0001 |
| U6KWI1 | Prolyl-tRNA synthetase, putative | 3.49 | 0.0001 |
| U6KJ69 | Uncharacterized protein | 3.45 | 0.0006 |
| U6KI46 | Hypoxanthine phosphoribosyltransferase | 3.34 | 0.0354 |
| H9B9L2 | Uncharacterized protein | 3.32 | 0.0058 |
| H9B9F9 | Uncharacterized protein | 3.22 | ＜0.0001 |
| U6KWP0 | YALI0F04037p, related | 3.22 | 0.0086 |
| U6KRD9 | Carbonic anhydrase domain containing protein, putative | 3.14 | 0.0002 |
| U6KY79 | DnaJ domain-containing protein, putative | 2.98 | 0.0009 |
| U6KKU0 | S-adenosylmethionine synthase | 2.93 | 0.0001 |
| U6L392 | Uncharacterized protein | 2.85 | 0.0018 |
| U6L7L3 | Uncharacterized protein | 2.84 | 0.0003 |
| U6KXP2 | Exportin, putative | 2.78 | 0.0259 |
| U6KQ11 | GTP-binding nuclear protein | 2.75 | ＜0.0001 |
| U6L4W6 | ABC transporter, putative | 2.72 | 0.0262 |
| U6KZN0 | Eukaryotic translation initiation factor 4a, putative | 2.68 | ＜0.0001 |
| U6L6M7 | DNA topoisomerase | 2.68 | 0.0127 |
| U6KGD0 | Uncharacterized protein | 2.67 | 0.0011 |
| U6L8U7 | Glycyl-tRNA synthetase, putative | 2.66 | 0.0006 |
| U6KN00 | Elongation factor 2, putative | 2.61 | ＜0.0001 |
| U6L6Z7 | Histidyl-tRNA synthetase, putative | 2.60 | 0.0141 |
| U6KW42 | Ribosomal protein L34, putative | 2.53 | 0.0004 |
| U6L1Q0 | Uncharacterized protein | 2.53 | 0.0002 |
| U6L816 | Phosphoglycerate kinase | 2.51 | 0.0001 |
| U6L658 | Mannose-6-phosphate isomerase, putative | 2.50 | 0.0010 |
| U6KYH6 | Alanine--tRNA ligase | 2.44 | 0.0033 |
| U6KSD4 | Eukaryotic peptide chain release factor subunit, putative | 2.44 | 0.0008 |
| U6KWS7 | DnaJ domain-containing protein, putative | 2.39 | 0.0002 |
| U6KYZ2 | Uncharacterized protein | 2.37 | ＜0.0001 |
| U6KN65 | Adenosylhomocysteinase | 2.37 | ＜0.0001 |
| H9B9C8 | Peptidylprolyl isomerase | 2.37 | 0.0004 |
| U6KHG0 | SAG family member | 2.36 | 0.0005 |
| D7RXM4 | Facilitative glucose transporter, putative | 2.35 | 0.0001 |
| U6KL00 | ATP-dependent RNA helicase, putative | 2.33 | 0.0472 |
| U6KHI2 | Uncharacterized protein | 2.33 | 0.0006 |
| A0A172WCE4 | Phosphotransferase | 2.33 | 0.0002 |
| U6KQV3 | Histone H2A | 2.32 | 0.0001 |
| U6KQA4 | Uncharacterized protein | 2.31 | 0.0003 |
| U6KUL8 | SAG family member | 2.31 | 0.0027 |
| U6KQM7 | Ubiquitin-conjugating enzyme e2, putative | 2.28 | 0.0001 |
| U6L627 | Rhomboid-like protease 5, putative | 2.23 | 0.0022 |
| U6KH97 | RNA binding protein, putative | 2.22 | 0.0004 |
| A0A184W4F4 | Elongation factor 2 | 2.22 | 0.0015 |
| U6KJR0 | AP-1 complex subunit gamma | 2.22 | 0.0335 |
| F4MKA7 | Immune mapped protein 1 | 2.21 | 0.0024 |
| U6L555 | Seryl-tRNA synthetase, putative | 2.19 | 0.0008 |
| U6KLS1 | Cysteine desulfurase, putative | 2.17 | 0.0002 |
| U6KWV4 | Opine dehydrogenase, putative | 2.17 | 0.0004 |
| U6KWW5 | Uncharacterized protein | 2.17 | 0.0030 |
| U6L120 | Uncharacterized protein | 2.13 | 0.0110 |
| U6KXG0 | ADP ribosylation factor 1, putative | 2.11 | 0.0008 |
| U6KMF8 | Prefoldin subunit, putative | 2.10 | 0.0353 |
| H9B971 | Uncharacterized protein | 2.10 | 0.0001 |
| U6KZ54 | Arginyl-tRNA synthetase, putative | 2.10 | 0.0018 |
| U6KFY1 | Triosephosphate isomerase | 2.06 | 0.0043 |
| U6L3E4 | 4-hydroxy-3-methylbut-2-en-1-yl diphosphate synthase, putative | 2.04 | 0.0002 |
| U6L8L6 | Chaperonin complex component, related | 2.01 | 0.0339 |
| U6L0E4 | Uncharacterized protein | 0.49 | ＜0.0001 |
| U6L2Z0 | Uncharacterized protein | 0.49 | 0.0082 |
| U6KS84 | Uncharacterized protein | 0.49 | 0.0003 |
| A0A1P7YD75 | Phosphodiesterase | 0.49 | 0.0013 |
| U6KG50 | RNA binding motif-containing protein, putative | 0.49 | 0.0052 |
| U6L8A7 | Uncharacterized protein | 0.49 | 0.0052 |
| U6KLF0 | Uncharacterized protein | 0.49 | 0.0339 |
| U6KQH6 | Uncharacterized protein | 0.49 | 0.0002 |
| U6KWQ6 | Dynein heavy chain protein, related | 0.48 | 0.0001 |
| H9B8Z9 | 40S ribosomal protein SA | 0.48 | 0.0001 |
| U6KW18 | CMGC kinase, putative | 0.48 | 0.0059 |
| U6L8V9 | Uncharacterized protein | 0.48 | 0.0002 |
| U6LBU5 | V-type proton ATPase subunit a | 0.48 | 0.0001 |
| U6KLM2 | Uncharacterized protein | 0.48 | 0.0001 |
| Q70CE1 | SAG family member (Sag7) | 0.47 | ＜0.0001 |
| U6KLD7 | Alkyl sulfatase, putative | 0.47 | 0.0026 |
| U6KHI8 | Uncharacterized protein | 0.46 | 0.0291 |
| U6KR42 | PT repeat family protein, related | 0.46 | ＜0.0001 |
| U6KQN3 | Uncharacterized protein | 0.46 | 0.0008 |
| U6L2Y4 | Uncharacterized protein | 0.46 | 0.0008 |
| U6KZG9 | Uncharacterized protein | 0.45 | 0.0016 |
| H9B912 | 60S acidic ribosomal protein P1, putative | 0.44 | 0.0001 |
| U6KZS4 | 40S ribosomal protein S28, putative | 0.44 | 0.0004 |
| U6KHZ9 | Uncharacterized protein | 0.44 | 0.0035 |
| Q70CC7 | SAG family member (Sag23) | 0.43 | ＜0.0001 |
| U6KSU0 | Uncharacterized protein | 0.43 | 0.0032 |
| U6L774 | Uncharacterized protein | 0.43 | 0.0001 |
| U6KX85 | Uncharacterized protein | 0.43 | 0.0001 |
| U6KQD1 | Uncharacterized protein | 0.43 | 0.0286 |
| U6KIB8 | Uncharacterized protein | 0.43 | ＜0.0001 |
| U6KYV2 | Uncharacterized protein | 0.42 | 0.0045 |
| U6L3R1 | SAG family member | 0.42 | ＜0.0001 |
| U6KR57 | SAG family member | 0.42 | 0.0001 |
| U6KP46 | Uncharacterized protein | 0.42 | 0.0009 |
| U6KJF8 | Lysophospholipase, putative | 0.41 | ＜0.0001 |
| U6KS65 | Uncharacterized protein | 0.40 | ＜0.0001 |
| U6LB52 | Uncharacterized protein | 0.39 | ＜0.0001 |
| H9B984 | Reticulon domain-containing protein, putative | 0.39 | 0.0145 |
| U6L607 | Uncharacterized protein | 0.38 | ＜0.0001 |
| U6L1H2 | Uncharacterized protein | 0.37 | 0.0097 |
| H9B926 | SAG family member | 0.37 | ＜0.0001 |
| H9B939 | SAG family member (Sag22) | 0.36 | ＜0.0001 |
| U6KUL4 | SAG family member | 0.35 | ＜0.0001 |
| Q70CD2 | SAG family member (Sag16) | 0.35 | ＜0.0001 |
| U6KY37 | Uncharacterized protein | 0.35 | 0.0169 |
| H9BA68 | SAG family member | 0.35 | 0.0001 |
| U6L625 | SAG family member | 0.35 | 0.0007 |
| Q70CC6 | SAG family member (Sag18) | 0.35 | ＜0.0001 |
| U6L2F6 | Uncharacterized protein | 0.34 | 0.0006 |
| Q70CD9 | SAG family member (Sag4) | 0.34 | ＜0.0001 |
| U6KXX5 | Alpha-1,4 glucan phosphorylase | 0.33 | ＜0.0001 |
| U6L273 | Uncharacterized protein | 0.33 | 0.0002 |
| U6L4L5 | Uncharacterized protein | 0.33 | ＜0.0001 |
| U6KSR6 | Ribosome biogenesis regulatory protein | 0.33 | 0.0282 |
| U6KYA9 | Uncharacterized protein | 0.33 | ＜0.0001 |
| U6KZU0 | Uncharacterized protein | 0.32 | ＜0.0001 |
| U6KLW6 | Uncharacterized protein | 0.32 | 0.0024 |
| Q70CC8 | SAG family member (Sag21) | 0.31 | ＜0.0001 |
| U6KQ59 | Uncharacterized protein | 0.31 | 0.0020 |
| H9B9V7 | Uncharacterized protein | 0.30 | 0.0048 |
| U6L4K6 | Uncharacterized protein | 0.30 | 0.0002 |
| U6L2E4 | Vacuolar ATP synthase catalytic subunit A, putative | 0.30 | 0.0456 |
| H9B975 | Uncharacterized protein | 0.28 | 0.0019 |
| H9B9H4 | SAG family member | 0.27 | 0.0004 |
| C8TDV9 | Uncharacterized protein | 0.25 | ＜0.0001 |
| U6KWQ3 | Uncharacterized protein | 0.25 | 0.0006 |
| U6L3P3 | DnaJ domain-containing protein, related, related | 0.24 | 0.0027 |
| U6KN45 | Dynein intermediate chain, putative | 0.23 | 0.0012 |
| U6KYS5 | Uncharacterized protein | 0.21 | 0.0001 |
| U6KID7 | Uncharacterized protein | 0.21 | ＜0.0001 |
| U6L4U5 | SAG family member | 0.20 | ＜0.0001 |
| U6L0K6 | Uncharacterized protein | 0.20 | 0.0265 |
| U6KVB1 | Uncharacterized protein | 0.19 | 0.0269 |
| U6L233 | SAG family member | 0.17 | 0.0009 |
| U6KTF6 | Kelch motif domain-containing protein, putative | 0.13 | 0.0379 |
| U6KIG0 | Selenophosphate synthetase, putative | 0.11 | 0.0262 |
| U6L620 | Uncharacterized protein | 0.08 | 0.0001 |
| Q70CD1 | SAG family member (Sag13) | 0.06 | ＜0.0001 |
| U6KGF5 | Uncharacterized protein | 0.05 | 0.0001 |

^a^ Access was protein accession in the UniProt *Eimeria tenella* database;

^b^ Fold change was quantitative expression difference of proteins in response to EZL;

^c^ *P-*value <0.05 identified the significant expression difference of SDPs in the E-group compared with those in the C-group; *P-*value < 0.0001 indicated that the exact *P-*value was very small.
